# Lista de Diagnósticos de Cardiopatias Congênitas da Classificação Internacional de Doenças 2011 (CID-11): Aspectos da Tradução para o Português

**DOI:** 10.36660/abc.20210143

**Published:** 2021-09-01

**Authors:** Vera Demarchi Aiello, Sandra da Silva Mattos

**Affiliations:** 1 Universidade de São Paulo Faculdade de Medicina Instituto do Coração, Hospital das Clínicas São Paulo SP Brasil Laboratório de Anatomia Patológica, Instituto do Coração, Hospital das Clínicas HCFMUSP, Faculdade de Medicina, Universidade de São Paulo, São Paulo, SP – Brasil; 2 Unidade de Cardiologia Materno-Fetal Real Hospital Português Recife PE Brasil Real Hospital Português, Unidade de Cardiologia Materno-Fetal, Recife, PE – Brasil

**Keywords:** Cardiopatias congênitas, Terminologia como Assunto, Classificação Internacional de Doenças (CID)

## Contexto

A Sociedade Internacional para Nomenclatura em Cardiologia Pediátrica e Cardiopatias Congênitas (ISNPCHD – International Society for Nomenclature of Pediatric and Congenital Heart Disease) é uma organização composta por especialistas em Cardiologia Pediátrica, Cirurgia Cardíaca e Morfologia. Ela foi criada com a missão de unificar a terminologia das doenças cardíacas congênitas e adquiridas na infância em todo o mundo. A nomenclatura unificada permite comparações diagnósticas interinstitucionais e internacionais e a troca de informação sobre os resultados do manuseio clínico-cirúrgico de crianças e adultos com doenças cardíacas congênitas.^1^

Iniciando com reuniões anuais, com o Grupo de Trabalho Internacional para Mapear a Nomenclatura e Codificação da Cardiopatia Congênita e Pediátrica”,^1^ os membros decidiram criar, em 2005, a Sociedade Internacional (ISNPCHD), estendendo a filiação para incorporar profissionais de áreas pouco representadas no mundo.

Ao longo dos anos, três grupos de trabalho foram criados na ISNPCHD: o Grupo de Trabalho das Definições, o Grupo de Trabalho de Mapeamento de Códigos (Grupo de Trabalho de Nomenclatura) e o Grupo de Trabalho dos Arquivos de Imagens.

As tarefas iniciais da Sociedade foram concluídas com o desenvolvimento do Código Internacional de Cardiopatias Congênitas e Pediátricas (IPCCC), uma lista de diagnósticos e procedimentos vinculados a um código numérico de seis dígitos, e com o mapeamento bidirecional entre os códigos da Associação Europeia para Cardiologia Pediátrica e Congênita e o Sistema de Nomenclatura do Banco de Dados do Projeto Internacional para Nomenclatura da Cirurgia Cardíaca de Cardiopatias Congênitas. O código numérico do IPCCC foi derivado do Código Europeu de Cardiologia Pediátrica e serviu como estrutura central para o processo de mapeamento.

As listas e códigos do IPCCC (em inglês) estão disponíveis gratuitamente para download no website da Sociedade: http://www.IPCCC.net.

Durante a preparação da 11ᵃ atualização do Código Internacional de Classificação de Doenças (CID-11) da Organização Mundial da Saúde (OMS), em 2007, os membros da ISNPCHD receberam a missão de revisar a lista de diagnósticos para cardiopatias congênitas e pediátricas da versão anterior do CID. Também foi solicitado que o grupo do ISNPCHD atualizasse a lista de acordo com o consenso da nomenclatura que havia sido construído entre os especialistas desde a criação do Comitê de Nomenclatura.^2^

Como o CID-9 e o CID-10 continham, respectivamente, apenas 29 e 73 termos dedicados às malformações congênitas do sistema circulatório, havia o propósito de expandir esse número de diagnósticos no sentido de cobrir as malformações cardiovasculares mais frequentes, o que resultou em mais de 300 termos na 11ᵃ versão do CID. As malformações muito raras não foram incluídas nesta nova lista do CID, porque, pela sua raridade, elas não precisam ser citadas em estatísticas vitais. Essas lesões muito raras (o “anel intravalvar mitral” é um exemplo entre vários outros) devem ser incluídas sob a categoria genérica do nível superior (“anomalia congênita da valva mitral”, no exemplo supracitado).

## Métodos

### A lista do CID-11 para cardiopatias congênitas (acesse o material suplementar no fim do artigo)

Iniciada oficialmente no Japão, em 2007, pelo menos uma das sessões anuais da ISNPCHD passou a ser dedicada à discussão da lista do CID-11, seguindo a hierarquia dos termos da IPCCC, já desenvolvida e atualizada pelo grupo que mapeou as duas principais listas diagnósticas utilizadas atualmente – aquelas da Sociedade Europeia de Cardiologia e da Sociedade dos Cirurgiões Torácicos –, como previamente explicado.

Além da organização em seis níveis hierárquicos, os termos foram mapeados para os códigos IPCCC e para o código alfanumérico do CID-10 anterior da OMS. Além disso, para cada um dos códigos, foram providenciadas uma definição e uma lista de sinônimos e abreviaturas comumente utilizadas.

Chegar a um consenso sobre as definições foi a parte do processo que trouxe as maiores discussões, além de ser fundamental, visto que o objetivo era proporcionar uma compreensão clara do diagnóstico morfológico para codificadores e usuários em todo o mundo.

A lista com 324 diagnósticos e seus respectivos códigos IPCCC foi apresentada no Encontro de Nomenclatura durante o Congresso Mundial de Cardiologia Pediátrica em Barcelona, em 2017, e publicada na revista médica Cardiology in the Young, no mesmo ano.^2^

O produto final foi, então, juntamente com a lista completa do CID-11, disponibilizado on-line pela OMS em 2018, sob “Anomalias do desenvolvimento/Anomalias do desenvolvimento estrutural afetando primariamente um sistema do corpo/Anomalias do desenvolvimento do sistema circulatório/Anomalias do desenvolvimento do coração e grandes vasos. A versão 11 do CID está programada para entrar em vigor em janeiro de 2022. Nesse ínterim, os países membros devem se preparar para a sua implementação, incluindo o trabalho de tradução, entre outras etapas.

Aconteceram três principais mudanças no sistema, incluindo um aumento na quantidade de capítulos. O novo sistema de códigos não é semelhante ao utilizado no CID-10, como discutido no documento acessado eletronicamente.

### Adição de novos termos à lista

Após a finalização da lista de termos de cardiopatias congênitas pela ISNPCHD, 94 novos termos foram acrescentados pelo staff da OMS. Esses termos estão sob análise de membros da ISNPCHD, que eventualmente decidirão sob a sua manutenção na lista do CID-11, os retirarão (por incorreção científica) ou os substituirão em outras seções do CID-11.

### Tradução da lista do CID-11 das cardiopatias congênitas para outras línguas

Como aparece na página da OMS/CID, “CID é uma padronização internacional e deve ser multilíngue”.

A prioridade para representação multilíngue do CID-11 serão os seis idiomas oficiais das Nações Unidas: inglês, francês, espanhol, russo, chinês e árabe.

Em uma tentativa de acelerar o uso dos termos das cardiopatias congênitas por equipes de saúde que não falam inglês, em 2019, Béland et al.^3^ prepararam e publicaram no Canadian Journal of Cardiology, a tradução francesa para a lista de cardiopatias congênitas do CID-11.

Em 2018, devido à ausência de uma tradução para a Língua Portuguesa, nós abraçamos a causa e apresentamos aqui o produto final.

### Alguns aspectos específicos da tradução do CID-11 para o português do Brasil

O Brasil é, nos dias atuais, o país do mundo com o maior número de pessoas que falam Português como sua língua nativa. O Português é, atualmente, a sexta língua falada no mundo pelo número de nativos, o que corresponde a 2,87% da população mundial.

Ainda assim, o Português não é considerado uma língua oficial pela OMS, e ainda não temos uma plataforma no site do CID.

Além disso, o número de cardiologistas pediátricos associados ao Departamento de Cardiologia Pediátrica da Sociedade Brasileira de Cardiologia, em 2020, ultrapassa 750, o que claramente demonstra a importância de se manter essa comunidade atualizada com a nomenclatura das cardiopatias congênitas.

Embora o processo de faturamento do Sistema Único de Saúde (SUS) do Ministério da Saúde esteja atualmente baseado na 10a versão do CID, a qualidade das melhorias incluídas na nova versão (CID-11), juntamente com a possibilidade de mapear os novos termos para as listas anteriores, evidenciam a importância de disponibilizar a nova lista em Português.

Além dos termos diagnósticos, as definições, os comentários e os sinônimos, que estão todos disponíveis na página do CID/OMS na internet, foram traduzidos para essa lista em Português.

Durante a tradução, algumas decisões foram tomadas para facilitar a compreensão e o processo de busca/resgate. A seguir, são mostrados alguns exemplos:

- Uns poucos termos diagnósticos foram mantidos em inglês, entre parênteses, após o termo em Português, devido ao seu uso generalizado pelos cardiologistas brasileiros (p. ex., straddling, criss-cross, sling).
- Alguns termos em latim foram mantidos também entre parênteses pelos mesmos motivos anteriores (p. ex., truncus, ostium primum).
- Manutenção de epônimos (p. ex., Fallot, Ebstein), assim como aparecem na lista em inglês.
- Não tradução de alguns termos em inglês, pois não fariam sentindo em Português (p. ex., upstairs-downstairs ventricle).
- Finalmente, porém não menos importante, faz-se necessário discutir o termo “comunicação” entre câmaras, como termo preferido nas línguas românticas como Português, Italiano, Francês e Espanhol, em vez do termo “defeito septal” comumente aplicado em Inglês e outras línguas germânicas. É fácil entender que nem todas as comunicações interatriais são causadas por um defeito na anatomia do septo interatrial – “o defeito do seio coronário” servindo como exemplo. Ao mesmo tempo, nem todas as comunicações interventriculares coincidem com o que chamamos de defeito septal ventricular.^4^ Especialmente quando há cavalgamento de um tronco arterial (p. ex., da aorta), a comunicação interventricular é o plano do espaço entre a valva aórtica e o topo do septo trabecular. Essa região permite a saída do fluxo do ventrículo esquerdo, mas o orifício que o cirurgião fecha, o qual chamamos comumente de comunicação interventricular, é a borda direita desse plano, como no caso da tetralogia de Fallot. Nessa tradução, decidimos manter “comunicação interventricular” em todos os locais em que o termo em inglês “ventricular septal defect” foi utilizado.

### Perspectivas futuras para os termos do CID-11 traduzidos para o português

Como mencionado previamente, no Brasil, atualmente, o SUS é a principal ferramenta utilizada pelo Ministério da Saúde para o processo de faturamento em hospitais públicos. Embora a implantação do CID-11 pelo SUS não deva acontecer em um futuro próximo, isso não deve limitar o acesso de profissionais que cuidam de crianças e adultos com cardiopatias congênitas ao sistema atualizado de sua nomenclatura.

A tradução que aqui apresentamos já foi submetida à OMS desde agosto de 2019. No entanto, fomos informados de que o trabalho ainda não foi incorporado pela indisponibilidade de uma plataforma em português no site da Organização. Nossa decisão de publicar a lista em uma revista de Cardiologia local, assim como uma versão eletrônica no site do Departamento de Cardiologia Pediátrica e Congênita da Sociedade Brasileira de Cardiologia, tem como objetivo dar acesso aos profissionais e serviços especializados ao novo sistema de nomenclatura e códigos, aumentando assim as suas capacidades para trocar informações a respeito de prevalência, tratamento e prognóstico das cardiopatias congênitas em países de Língua Portuguesa.

O próximo passo neste processo está sendo o desenvolvimento de um aplicativo para mapear CID-10, CID-11 e IPCCC, pois, caso o sistema oficial não seja atualizado em relação ao processo de faturamento, ainda assim, cardiologistas e cirurgiões pediátricos poderão utilizar o sistema de codificação atualizado, em paralelo, na sua prática diária.

### O processo da tradução

A ideia de realizar esta tradução surgiu a partir de uma conversa informal entre as autoras, e a decisão foi acelerada por uma visita fortuita de uma das autoras (VDA) na última semana de 2018, à bela cidade do Recife, onde a mora a outra autora (SSM). Encorajadas pela visão deslumbrante do Oceano Atlântico no Nordeste do Brasil, nos encontramos tête-à-tête durante cinco dias iniciais, e completamos a tarefa em reuniões on-line até meados de 2019, mesmo antes de esse tipo de atividade se tornar prática corriqueira como agora, durante a era da Covid-19.

Após a tradução, a lista foi checada para consistência e correção gramatical por um profissional da área.

Esperamos que o resultado de nosso esforço seja bem recebido pela comunidade científica dos países de Língua Portuguesa e que facilite a troca de informações entre as inúmeras instituições de saúde nesses países, de modo a promover um suporte para pesquisa com foco no bem-estar de pacientes portadores de cardiopatias congênitas.
