# Supplementary material for: Lista de Diagnósticos de Cardiopatias Congênitas da Classificação Internacional de Doenças 2011 (CID-11): Aspectos da Tradução para o Português
Source: Arq Bras Cardiol. 2021 Sep 1;117(3):558–60. [Article in Portuguese] doi: 10.36660/abc.20210143 (PMC8462966; doi:10.36660/abc.20210143)
Supplement: Translation

# The Congenital Heart Disease Diagnosis List for the eleventh iteration of the International Classification of Diseases (ICD11): Translation to Brazilian Portuguese

**Authors:** Vera Demarchi Aiello, Sandra da Silva Mattos

**Affiliations:** 1 São Paulo SP Brazil Laboratório de Anatomia Patológica, Instituto do Coração, Hospital das Clínicas HCFMUSP, Faculdade de Medicina, Universidade de São Paulo, São Paulo, SP – Brazil; 2 Recife PE Brazil Real Hospital Português, Unidade de Cardiologia Materno-Fetal, Recife, PE – Brazil

**Keywords:** Heart Defects Congenital, Terminology as Topic, International Classification of Diseases

## Background

The International Society for Nomenclature of Pediatric and Congenital Heart Disease (ISNPCHD) is an organization composed by specialists in Pediatric Cardiology, Cardiac Surgery and Morphology. It was created aiming at unifying the terminology of congenital and acquired pediatric heart disease across the world. The unified lexicon allows for inter-institutional and international diagnostic comparisons and exchange of information on the outcomes of children and adults with congenital heart defects^1^.

Initially gathering annually, as the “The International Nomenclature Working Group for the Mapping and Coding of Nomenclatures for Pediatric and Congenital Heart Disease”,^1^ the members decided to create, in 2005, the International Society (ISNPCHD), extending the membership to incorporate professionals from underrepresented areas of the world.

Over time, three working groups of the ISNPCHD were created: the Definitions Working Group,^1^ the Mapping and Coding Group (or Nomenclature Working Group) and the Archiving Working Group.

The initial tasks of the Society were accomplished with the development of the International Pediatric and Congenital Cardiac Code (IPCCC), a list of diagnoses and procedures, each linked to a six-digit numeric code, and the bidirectional cross-mapping between “ The European Paediatric Cardiac Code of the Association for European Paediatric and Congenital Cardiology” and “ The nomenclature system of the International Congenital Heart Surgery Nomenclature and Database Project ”. The IPCCC numerical code was derived from “The European Paediatric Cardiac Code” and served as the backbone for the mapping process.

The IPCCC codes and lists (in English) are freely available for download from the Society’s website at http://www.IPCCC.net.

During the preparation of the eleventh iteration of the International Classification of Diseases (ICD11) of the World Health Organization (WHO), in 2007 the members of the ISNPCHD were given the mission of revising the existing list of diagnoses for pediatric and congenital heart disease from the previous ICD version. The ISNPCHD group was also expected to update the list according to the nomenclature consensus that has been built among specialists since the creation of the Nomenclature Committee.^2^

While the ICD9 and ICD10 versions had respectively only 29 and 73 terms devoted to the congenital malformations of the circulatory system, the intention was to expand the number of diagnoses in order to cover most of the frequent cardiovascular malformations, which resulted in more than 300 terms in the 11^th^ version of the ICD. Of course, the rarest lesions were not included in the new ICD list, because their uncommon occurrence is not worth reporting in vital statistics. Such rare lesions (among many others, “intravalvar mitral ring” is an example) should be included under the generic upper level category (“congenital anomaly of the mitral valve”, in the abovementioned example ).

## Methods

### The ICD11 list for Congenital Heart Defects (access the supplementary material at the end of the article)

Officially starting in Japan, 2007, at least one session of each ISNPCHD annual meeting was devoted to the discussion of the ICD11 list, following the hierarchy of terms from the IPCCC, already developed and maintained by the group and mapped to the two main diagnosis’ lists currently in use, the ones from the European Society of Cardiology and the Society of Thoracic Surgeons, as previously explained.

Besides the organization of the list into six hierarchical levels, the terms were mapped to the IPCCC code and to the previous ICD10 alphanumeric code of the WHO. Also, a definition and a list of synonyms and commonly used abbreviations were provided for each diagnosis.

Reaching a consensus on the definitions was the most contentious part of the process and of fundamental importance, since the goal was to provide a clear comprehension of the morphological diagnosis to coders all over the world.

The list of 324 diagnoses with their respective IPCCC codes were presented at the Nomenclature Summit of the World Congress of Pediatric Cardiology in Barcelona, 2017 and published in the journal ‘Cardiology in the Young’ in the same year.^2^

The final product was then, together with the complete ICD11 list, released by the WHO in 2018 (https://icd.who.int/dev11/f/en), under “Developmental anomalies / Structural developmental anomalies primarily affecting one body system / Structural developmental anomalies of the circulatory system / Structural developmental anomaly of the heart or great vessels”. The 11^th^ version is meant to be implemented in January 2022. Meanwhile, the country members will prepare for its implementation, including the translation work, etc. There have been many major changes in the system, including an increase in the number of chapters. The new coding system is not similar to the one used in the ICD10, as discussed in the document accessed electronically on October 19, 2020: https://www.icd10monitor.com/icd-11-hurry-up-and-wait.

### Addition of new terms to the list

After the completion of the congenital heart disease list of terms by the ISNPCHD, 94 new terms were added by the WHO staff. These terms are currently under analysis by the ISNPCHD members, who will eventually decide regarding their maintenance in the ICD11 list, their removal (because of scientific incorrectness) or replacement in other sections of the ICD11.

### Translation of the ICD11 list for Congenital Heart Defects to other languages

As it appears at the WHO/ICD webpage, “ICD, as an international standard, has to be multilingual.”

The priority for multilingual representation of the ICD11 will be the 6 Official United Nations Languages: English, French, Spanish, Russian, Chinese, Arabic.

In an initiative aimed at accelerating the use of ICD11 congenital heart disease terms by non-English speaking Health teams, in 2019 Béland et al.^3^ prepared and published the French translation of the ICD11 list for congenital heart defects in the Canadian Journal of Cardiology.

In 2018, given the lack of a translation to the Portuguese language, we embraced the task, and the final product is presented here.

### Some particular aspects of the translation of the ICD11 terms into Brazilian Portuguese

Brazil is currently the country in the world with the largest number of Portuguese-speaking people as their native language. Portuguese is currently the sixth language by number of native speakers, which comprises 2.87% of the world’s population

(Wikipedia, accessed in October 19^th^, 2020: https://en.wikipedia.org/wiki/List_of_languages_by_number_of_native_speakers).

Nevertheless Portuguese is not considered an official language by the WHO (WHO webpage, accessed on October 19, 2020: https://www.who.int/about/who-we-are/multilingualism) and still does not have a platform at the ICD site.

Moreover, the number of Pediatric Cardiologists associated to the Department of Pediatric Cardiology of the Brazilian Society of Cardiology in 2020 surpasses 750, what clearly highlights the importance of maintaining this community updated with the nomenclature of congenital heart defects.

Although the billing process of the Unified Health System (SUS) of the Brazilian Ministry of Health is currently based on the 10^th^ version of the ICD, the quality of the improvements included in the new version (ICD11), together with the possibility of mapping the new terms to the previous ICD list, makes it fundamental to make the new Portuguese list available.

Not only the diagnostic terms, but definitions, commentaries and synonyms, which are all available at the ICD/WHO website, were translated in this new list, as well.

During the translation, some decisions were made in order to facilitate the comprehension and the search/retrieval process. Some examples are:

- A few diagnostic terms were maintained in English, in parenthesis after the term in Portuguese, due to their widespread use among the Brazilian cardiologists (examples: crisscross, sling).
- Maintenance of Latin terms when of common use, similarly to the previous decisions (examples: truncus, ostium primum).
- Maintenance of eponyms (Fallot, Ebstein) as they appear in the English version of the diagnosis list.
-Non-translation of some English terms, since they make no sense in Portuguese (example: upstairs-downstairs ventricle).
- Last but not least, we have to discuss the use of the word “communication” between chambers as the most preferred term in Romance languages like Portuguese, Italian, French and Spanish, over the term “septal defect” commonly applied in English and other Germanic languages. It is easy to understand that not all interatrial communications are caused by defects within the anatomic atrial septum, with “coronary sinus defect” being an example. Likewise, not all the interventricular communications coincide with what we call a ventricular septal defect.^4^ Especially when there is overriding of an arterial trunk, the interventricular communication is the plane of space between the overriding valve and the top of the trabecular septum. This area is the outflow for the left ventricle, but the hole closed by the surgeon, what we usually call the ventricular septal defect, is the right border of this plane, as in Tetralogy of Fallot. In our translation, we decided to maintain “comunicação interventricular” (interventricular communication) in all the places where the term “ventricular septal defect” was found.

### Future perspectives for the translation of ICD11 terms into Portuguese

As previously mentioned, in Brazil the Unified Health Insurance System (SUS) is currently the main tool used by the Ministry of Health for the billing process in public hospitals. Although the implantation of ICD11 by the Government does not seem to be on the horizon in the short or middle term in our country, we should not hinder the access of professionals who care for children and adults with congenital heart disease to an updated system of nomenclature of cardiac defects in Portuguese.

We submitted our translated list of terms to the WHO in 2019. However, we were recently informed that our work has not yet been incorporated due to the lack of a Portuguese platform in their website. Our decision to publish the list, in a local Journal of Cardiology and also electronically on the website of the Brazilian Society of Cardiology/ Department of Pediatric Cardiology, aims to give access to specialized professionals and services to the new coding system and nomenclature, thus enhancing their capabilities to exchange data about prevalence, treatment and prognosis of congenital heart disease in Portuguese-speaking countries.

As the next step in this process, we plan to develop an application to cross-map the ICD10 and ICD11 codes and the IPCCC, so that even if the official system does not change regarding billing purposes, the Pediatric Cardiologists and Surgeons can have a parallel and updated coding system to be used in daily practice.

### The process of translation

It all started during a conversation between the authors, and the decision was accelerated by the fortuitous visit of one of us (VDA) to the beautiful city of Recife - where the other one of us (SSM) lives - during the last week of 2018. Encouraged by the astonishing view of the Atlantic ocean in Northeast Brazil, we met tête-à-tête during 5 days, and completed the task during computer meetings along the year of 2019, even when virtual conferences were not yet a common practice as they are now in the COVID19 pandemic era.

After the translation, the list was checked for consistency and grammatical corrections by a professional in the area.

We do hope the result of our effort is welcomed by the Portuguese-speaking scientific community and that it can facilitate the exchange of data between the numerous health institutions in the country, thus providing background to research focused in the wellbeing of our patients with congenital heart defects.
